# The central role of natural killer cells in preeclampsia

**DOI:** 10.3389/fimmu.2023.1009867

**Published:** 2023-02-14

**Authors:** Xiaoqi Wei, Xiuhua Yang

**Affiliations:** Department of Obstetrics, The First Hospital of China Medical University, Shenyang, China

**Keywords:** preeclampsia, NK cells, pregnancy, immune, placenta

## Abstract

Preeclampsia (PE) is a disease that is unique to pregnancy and affects multiple systems. It can lead to maternal and perinatal mortality. The precise etiology of PE is unclear. Patients with PE may have systemic or local immune abnormalities. A group of researchers has proposed that the immune communication between the fetus and mother is primarily moderated by natural killer (NK) cells as opposed to T cells, since NK cells are the most abundant immune cells in the uterus. This review examines the immunological roles of NK cells in the pathogenesis of PE. Our aim is to provide obstetricians with a comprehensive and updated research progress report on NK cells in PE patients. It has been reported that decidual NK (dNK) cells contribute to the process of uterine spiral artery remodeling and can modulate trophoblast invasion. Additionally, dNK cells can stimulate fetal growth and regulate delivery. It appears that the count or proportion of circulating NK cells is elevated in patients with or at risk for PE. Changes in the number or function of dNK cells may be the cause of PE. The Th1/Th2 equilibrium in PE has gradually shifted to an NK1/NK2 equilibrium based on cytokine production. An improper combination of killer cell immunoglobulin-like receptor (KIR) and human leukocyte antigen (HLA)-C may lead to insufficient activation of dNK cells, thereby causing PE. In the etiology of PE, NK cells appear to exert a central effect in both peripheral blood and the maternal-fetal interface. To maintain immune equilibrium both locally and systemically, it is necessary to take therapeutic measures directed at NK cells.

## 1 Introduction

Preeclampsia (PE) is a disease that is unique to pregnancy and affects multiple systems. It can lead to maternal and perinatal mortality (1). Its incidence rate is approximately 2–8% (2). PE is referred to as a “Great Obstetrical Syndrome” (3). Approximately 50,000–60,000 pregnant women worldwide die annually from PE (4, 5). PE is defined as hypertension (systolic blood pressure ≥ 140mmHg and/or diastolic blood pressure ≥ 90mmHg) that occurs for the first time after twenty weeks of gestation and may include one or more of the following manifestations: proteinuria (≥ 300mg/day), maternal organ damage (liver, kidney, or nervous system), blood system involvement (such as thrombocytopenia), or evidence of placental dysfunction (such as fetal growth restriction and/or abnormal uterine artery Doppler) (6–8). Severe PE is characterized by significantly elevated blood pressure (≥160/110mmHg), proteinuria, pulmonary edema, severe neurological symptoms, liver and kidney dysfunction, or low platelets (9). PE can gradually progress to eclampsia, accelerated hypertension, acute renal failure, liver subcapsular hematoma, liver rupture, and heart failure, and can also result in placental abruption and intrauterine fetal death (10). Furthermore, the risk of developing cardiovascular illness is twelve times higher in females who have experienced PE, suggesting a link between PE and the development of cardiovascular disease (11). Early-onset PE (occurring prior to 34 weeks) and late-onset PE (occurring after 34 weeks) are subtypes of PE. For many years, the most effective treatment for PE has been termination of pregnancy. However, since it is often necessary to prematurely deliver the fetus and placenta, the prognosis for newborns is poor (12). Therefore, it is essential to investigate the pathogenesis of PE and implement effective treatments to improve the prognosis of mothers and infants.

PE can be caused by genetic factors, vascular endothelial cell damage, angiogenesis disorders, decreased invasion of trophoblasts, and insufficient recasting of uterine spiral arteries (13, 14). However, the precise etiology of PE is unclear. Research has demonstrated that patients with PE may have systemic or local immune abnormalities (15–17). Both the innate and the adaptive immune systems, such as neutrophils, monocytes, natural killer (NK) cells and T cells, contribute to the onset and progression of PE (18, 19). The two-stage theory of PE is gradually gaining acceptance. The first stage is characterized by poor placental formation in the first trimester, while the second stage is characterized by placental oxidative stress in late pregnancy. The first stage typically occurs prior to the onset of clinical symptoms and is characterized by impaired spiral artery remodeling, insufficient blood flow at the maternal-fetal interface and placental ischemia. The second stage typically manifests as a systemic inflammatory response in leukocytes and endothelial cells of the blood vessels.

Some researchers have hypothesized that the mother must acquire immune tolerance to the semi-allogeneic fetus (20). However, recently, this theory’s validity has been called into question. It is now believed that the maternal-fetal immune interaction is in an activated state and that the immune system at the implantation site should be activated rather than inhibited. Additionally, the immune homeostasis at the materno-fetal interface is functional and finely regulated in healthy pregnancies (21). A new theory regarding the immunological etiology of PE has been proposed by a few researchers. They believe that the primary mode of immune communication between the fetus and mother is moderated by NK cells rather than T cells, as NK cells are the most abundant immune cells in the uterus (22). Furthermore, CD56 cells in maternal peripheral blood begin to increase from the 12th week of pregnancy, while T cells begin to change until late pregnancy (22). Fragments of syncytiotrophoblast also support the theory that NK cells, rather than T cells, are the predominant cell type in PE. These fragments are released into the maternal blood circulation during the normal replacement and renewal of the placental surface (22). In PE patients, these debris are found in increased quantities (23). It is also worth noting that in human placental tissues, there is no major histocompatibility complex (MHC) on the surface of syncytiotrophoblast, except for the soluble form of human leukocyte antigen (HLA)-G (24). This means that it cannot stimulate the antigen response of maternal T cells (22). On the other hand, NK cells can regulate the inflammatory response by both promoting and inhibiting inflammation (22). As a result, NK cells play a crucial role in PE. Our aim is to provide obstetricians with a comprehensive and updated research progress report on NK cells in PE patients.

## 2 NK cell biology

NK cells have cytotoxic effects on both tumor and infected cells and are important to the innate immune system. NK cells are classified as CD3^-^CD56^+^ lymphocytes in humans. They account for about 10% of the total peripheral blood lymphocytes and can be distributed into two subtypes according to CD56 and CD16 expression. Approximately 90% of all peripheral blood NK (pNK) cells are CD56^dim^CD16^+^, mainly playing a cytotoxic role. In contrast, CD56^bright^CD16^-^ NK cells have little cytotoxic nature. They secrete a group of cytokines, such as granulocyte macrophage colony stimulating factor (GM-CSF), interferon-γ (IFN-γ), transforming growth factor-β (TGF-β), interleukin-10 (IL-10) and IL-13 (25–28). CD16 has the ability to stimulate antibody-dependent cell-mediated cytotoxicity (ADCC). Therefore, the decreased CD16 expression confers greater regulatory functions and diminished cytotoxic properties to uterine NK (uNK) and decidual NK (dNK) cells. The cytotoxicity of NK cells is modulated by three factors. Firstly, NK cells go through an education procedure, and only self-identified NK cells are cytotoxic (29). Moreover, these cytotoxic NK cells are influenced by a complex network that includes intricate communication between target cells and activating or inhibiting receptors on the surface of NK cells. Lastly, NK cytotoxicity is lower in the resting state than in the “primed” state. In contrast to the phenotype of pNK cells (CD56^dim^CD16^+^), uNK and dNK cells have a higher CD56 density and less CD16 expression, existing as CD56^bright^CD16^-^ NK cells, which show a phenotype of tissue resident NK (trNK) cells (30).

Because of the presence of diverse cell receptors on NK cells, the cytotoxic effect and cytokine production of these cells varies (31). NK cell receptors include killer cell immunoglobulin-like receptors (KIRs), the C-type lectin heterodimer family (CD94/NKG2), the Ly49 homodimers, the NK cytotoxicity receptors (NCRs), and the immunoglobulin-like transcripts (ILTs) (32, 33). The NK receptors repertoire can also be divided into two classes, activating and inhibitory receptors. The activating receptors consist of KIRs (KIR2DS1, KIR2DS2, KIR2DS4, KIR2DS5, KIR3DS1), C type lectin receptors (NKG2D, CD94/NKG2C, CD94/NKG2E/H), NCRs (NKp30, NKp44, NKp46), and immunoglobulin (Ig)-like receptors (2B4) (18). On the other hand, the inhibitory receptors include KIRs (KIR2DL1, KIR2DL2/3, KIR2DL5, KIR3DL1, KIR3DL2, KIR3DL7), C-type lectin receptors (CD94/NKG2A, CD161), Ig-like receptors (ILT-2), and leukocyte inhibitory receptors (LAIR-1) (18). CD56^dim^ NK cells express KIRs. However, the CD56^bright^ subgroup lacks KIRs but expresses a greater number of CD94/NKG2A receptors. The CD94/NKG2 receptors are MHC class I specific receptors and contain inhibitory CD94/NKG2A and activating CD94/NKG2C receptors. The NCRs include NKp30, NKp44, and NKp46. NKp46 and NKp30 are simultaneously expressed. Moreover, the NKp44 expression rises when NK cells are activated. NK cells interact with MHC antigens *via* a vast array of distinct receptors. HLA-E on extravillous cytotrophoblast (EVT) inhibits the trophoblast killing by interacting with CD94/NKG2A (34). HLA-G combines with KIR2DL4 receptors on NK cells to produce cytokines, chemicals, and angiogenic elements that stimulate trophoblast invasion and vascular development during embryo implantation. The communication between dNK cells and trophoblast HLA-C is of particular interest. Paternity partially affects the binding between trophoblast cells and dNK cells due to the polymorphism of HLA-C and KIR2D receptors.

Programmed death 1 (PD-1) is an immune checkpoint that limits immune activity by transmitting an inhibiting signal to T cells, thus participating in immunologic suppression in maternal-fetal tolerance (35). The expressions of PD-1 ligands (PD-L1 and PD-L2) have been confirmed in human decidual tissues (36). A recent study showed that PD-1 was also expressed by NK cells (37). The expression of PD-L1 from decidual CD56^+^ NK cells of normal pregnant women in the first trimester was significantly higher than that in peripheral blood (38). This indicates that PD-1/PD-L1 pathway is involved in maintaining immune homeostasis at the maternal-fetal interface in the first trimester of human pregnancy. In another mice experiment, researchers detected the expression of PD-1 by dNK cells from pregnant BALB-c mice at two weeks of gestation (39). Their findings showed that the expression level of PD-1 by dNK cells was significantly increased compared with that by pNK cells (39). Since the study was carried out on 14.5 days of pregnancy in mice, it is similar to the third trimester in human pregnancy. Indeed, PD-1 receptor was recently identified on dNK cells in the third trimester of human pregnancy (40). Moreover, with the development of pregnancy, the expression of PD-1 on dNK cells increases gradually (40). Therefore, this NK inhibitory receptor may play a crucial role in pregnancy, which needs further investigation.

The origin of dNK cells is not entirely established. Currently, there are three theories. Some authors hypothesized that the recruitment of pNK cells into the uterus causes dNK cell aggregation (41). Since pNK cells and dNK cells exhibited similar chemokine receptor models, including extremely low levels of CCR1 and CCR5 when they contacted decidual stromal cells (41). The second theory proposes that certain precursor dNK cells develop and mature gradually from endometrial NK (eNK) cells in response to pregnancy-related elements including IL-15 and progesterone (42, 43). According to the third theory, dNK cells could differentiate gradually from the hematopoietic precursor cells in decidua under the influence of unique stromal elements in the decidual microenvironment (44). Therefore, the dNK cell origin is complex, and the aforementioned three theories could coexist, indicating that additional research is required.

In the menstrual cycle, the number of eNK cells fluctuates in non-pregnant females. uNK cells make up approximately 20% of endometrial leucocytes in the proliferative stage and 40-50% in the secretory stage in the endometrium of non-pregnant females (45). A small number of agranular NK cells can be observed in the endometrium before ovulation and in the proliferative (follicular stage) of the menstrual cycle (46). Under the influence of progesterone, the proportion of these cells increased significantly after ovulation. However, when progesterone levels decrease, these cells die two days ahead of menstruation (28). During the mid-to-late secretory (luteal) phase, they rapidly increase and become granular, encircling the spiral arteries and endometrial glands (47). When uNK cells disappear prior to menstruation, the expression of soluble factors that maintain vascular integrity decreases, resulting in the monthly shedding of endometrium that initiates the menstrual cycle, indicating that uNK cells are included in endometrial renewal, differentiation, and decomposition (48).

During a normal pregnancy, the proportion of pNK cells increases in early pregnancy, decreases in middle pregnancy, and then further decreases in late pregnancy (49). The same pattern of NK cytotoxicity is observed in pregnancy (50). In early pregnancy, dNK cells constitute 70-80% of decidual leukocytes with the highest expression level (51). The focus is on whether the phenotype and importance of dNK cells vary in pregnancy. It is widely known that decidual CD16^+^ NK cells are lower in late pregnancy than in early pregnancy (52). However, the expression level of CD16^+^ dNK cells at term was greatly higher than that in early pregnancy (53). In the third trimester of near-term pregnancy, 40% of decidual lymphocytes are CD56^+^ NK cells (54). It was not different between the first and second trimesters in the number of CD56^+^ cells in the decidua basalis (placental bed containing EVTs), same as the decidua parietalis (non-placental bed without trophoblast cells) (55). However, the number of CD56^+^ NK cells decreased greatly in the third trimester (55). The percentage of CD56^+^ NK cells expressing perforin and granzyme B in placental bed biopsies at 16 to 20 weeks of gestation was significantly lower than at 8 to 10 and 12 to 14 weeks of gestation (56). In addition, there was no difference between the first and second trimesters in the number and expression level of cytokines secreted by dNK cells (57). Compared to the first trimester, dNK cells in the second trimester had more activating receptors, such as NKG2D and NKp80, but their degranulation capacity decreased significantly (57). In addition, other researchers discovered that the dNK cell number in CD45^+^ cells during full-term pregnancy was significantly lower, but its degranulation ability was significantly higher than in early pregnancy (58). The same team also discovered that dNK cells had fewer HLA-C-binding receptors, including KIR2DS1, KIR2DL2/3, and KIR2DL1, but more HLA-E-binding NKG2A receptors than those in the first trimester (58). In full-term pregnancy, dNK cells have distinct receptors and gene expression profiles, indicating that they are a special kind of NK cells. However, the dNK cell function in full-term pregnancy is unknown. Previous research has demonstrated that dNK cells gradually decrease beginning in the second trimester and almost completely disappear in term pregnancy (28). The diversity of results may be attributable to the various detection methods. Previous experiments determined the presence of NK cells through coloring the cytoplasmic granules. Granular leukocytes are only a few in decidua after 20 weeks of gestation (56). In subsequent tests, however, the distribution of NK cells was analyzed using CD56 immunostaining. In the third trimester, there are still a small number of agranular CD56^+^ dNK cells that are usually ignored. Consequently, dNK cells may undergo both functional and phenotypic changes at various stages of pregnancy.

## 3 Role of dNK cells in reproduction

### 3.1 Modulating spiral artery remodeling

**Figure 1:**
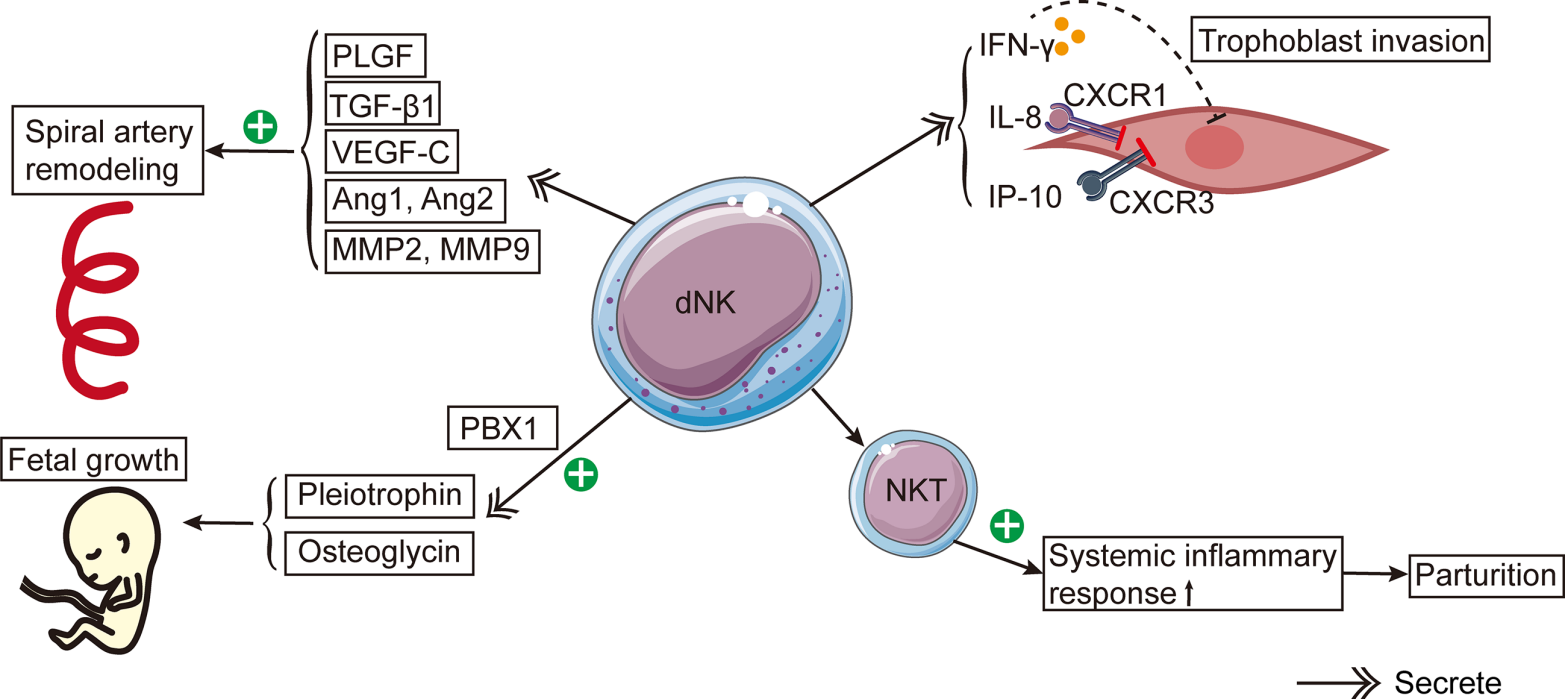
The schema of roles of dNK cells in reproduction. dNK cells contribute to the spiral artery remodeling by producing PlGF, TGF-β1, VEGF-C, Ang1, Ang2, MMP-2, and MMP-9. Besides, dNK cells secrete IFN-γ, IL-8, and IP-10, which inhibit trophoblast invasion. CXCR1 and CXCR3 are the corresponding receptors of IL-8 and IP-10, respectively. dNK cells stimulate fetal development by secreting pleiotrophin and osteoglycin. PBX1 raises the mRNA expression levels of pleiotrophin and osteoglycin. The activation of NKT cells induces a systemic inflammatory response, leading to the onset of parturition.

Reproduction-related dNK cell functions can be widely found in the existing studies (**Figure 1**). The process of spiral artery remodeling was divided into two stages. In the initial phase, the myometrium loses its elasticity and the endothelial cell layer ruptures (59). In the second stage, endovascular trophoblast cells enter the spiral arteries and take the place of the decidual endothelium and a portion of the myometrium (59). dNK cells contribute to the two phases of spiral artery remodeling (59–61). How dNK cells influence the spiral artery remodeling is unclear. Nevertheless, dNK cells secrete some factors, including placental growth factor (PlGF), TGF-β1, vascular endothelial growth factor-C (VEGF-C), angiopoietin 1 (Ang1), Ang2, matrix metalloproteinases-2 (MMP-2), and MMP-9 (61–63). These factors all participate in spiral artery remodeling (61, 62, 64). Moreover, dNK cells can interact with specific HLA ligands on trophoblast cells and indirectly contribute to the trophoblast-mediated recasting of spiral arteries (28).

Experiments with mice yielded an abundance of knowledge about the function of dNK cells in the spiral artery recasting in a healthy pregnancy. Similar to humans, the dNK cells in mice are stimulated (65) and produce numerous factors, such as IFN-γ, IL-22 (66, 67), PlGF (67) and VEGF (68). dNK cells in mice can express Ly49 receptors similar to human KIRs (69). In NK cell-deficient mice, the remodeling of spiral arteries is defective, and the structure of the decidua and myometrium is abnormal in the second trimester (70). Spiral artery remodeling was enhanced when NK cells were restored to NK cell-deficient mice (71). Similarly, IFN-γ administration to NK cell-deficient mice restored spiral artery remodeling, indicating that IFN-γ is important to spiral artery remodeling (66). Besides, IFN-γ may act on the initial stage of spiral artery remodeling (66). In addition, other factors are involved in the remodeling of spiral arteries in mice, including nitric oxide (72) and VEGF (73).

### 3.2 Regulating trophoblast invasion

Maternal dNK cells surround trophoblasts and regulate trophoblast invasion in a dose-dependent pattern (74). In the first trimester, dNK cells are a primary source of IFN-γ in the decidua, and IFN-γ may inhibit the invasion of EVT (62). It has been discovered that in human purified infiltrating trophoblasts, dNK cells could secrete IL-8, IFN-inducible protein (IP-10), and their corresponding receptors CXCR1 and CXCR3 (46). Experiments conducted *in vitro* revealed that after the addition of neutralizing antibodies IL-8 and IP-10, the capacity of dNK cells to induce trophoblast migration was significantly less than that of pNK cells (46). In brief, these experiments showed that IL-8 and IP-10 produced by dNK cells will inhibit trophoblast invasion (75).

Since mice are not ideal models for deep trophoblast incursion, rats are often used to explore the relationship between dNK cells and trophoblast invasion (76, 77). On the 15th, 17th and 20th day of pregnancy in rats, serial sections of the mesometrial triangle (similar to the human placental bed) were made, and the dNK cells were stained (43). It was discovered that dNK cells disappeared from the myometrium following EVT invasion, indicating that dNK cells modulate EVT invasion (43). dNK cells can also control the invasion of endovascular trophoblasts, as the invasion of endovascular trophoblasts increased on day 13.5 of pregnancy following the removal of NK cells in the first trimester (78). It is noteworthy that different species have distinct NK cell types. Methodologically, there is currently no model that can eliminate dNK cells only or eliminate this cell population by targeting specific antibodies.

### 3.3 dNK cells in fetal development

dNK cells could stimulate fetal growth and development. Prior to successful placentation in humans and mice, the CD94a^+^ subtype of dNK cells stimulated fetal growth by producing development-improving elements such as osteoglycin and pleiotrophin (79). The lack of these factors may lead to fetal bone dysplasia and growth restriction in mice (79). It has been revealed that the transcription factor PBX homeobox 1 (PBX1) can increase the mRNA expression levels of growth-promoting elements in dNK cells, thereby promoting fetal development in murine pregnancy models (80).

### 3.4 dNK cells in parturition

In the third trimester, dNK cells become agranular in the cytoplasm, suggesting that their function changes and they gradually prepare for delivery (55). dNK cells have been reported to regulate delivery. Using single cell RNA-sequencing to compare the basal plate, placental villous tissues, and chorioamniotic membranes of full-term and preterm pregnant women, researchers discovered that these tissues contained many lymphocytes, including T cells and NK cells (81). Moreover, compared to full-term pregnant women without labor, the single-cell signatures of NK cells were significantly elevated in full-term pregnant females with spontaneous delivery (81).

NKT cells are a small subpopulation of T cells that express certain surface markers on the NK cell surface. NKT cells can activate NK cells by producing IFN-γ. Even though only 0.01%- 0.1% of peripheral blood lymphocytes are NKT cells, their number in decidua is 10 times that of peripheral blood (82). Experiments *in vivo* revealed a remarkable rise in the activated NKT cell number in the decidua of preterm pregnant mice, indicating that the stimulation of NKT cells caused preterm birth by promoting systemic inflammatory response (83). However, the specific mechanism by which dNK cells cause delivery initiation is still unclear.

Collectively, dNK cells perform many important functions including modulating spiral artery remodeling, regulating trophoblast invasion, promoting fetal development and the initiation of labor in pregnancy. However, it has been revealed that dNK cells vanish by the end of the pregnancy (55, 58), therefore, dNK cells that play a role may have specific phenotypes or dNK cells in early pregnancy.

## 4 pNK cells in PE

Changes in pNK cells may occur in patients with PE, but the direction and magnitude of these changes are controversial (**Table 1**). According to the scientific literature, the cytotoxicity of pNK cells in PE patients may be higher (18, 85, 91, 92, 94–96), lower (97), or equal (84) to that of normal controls. The level of CD56^+^/NKp44^+^ cells in females with PE was significantly higher than that of healthy pregnant women at 12-20 weeks of gestation (18). The increased percentage of pNK cells may indicate the correspondingly elevated dNK cells, which may aid in identifying asymptomatic PE patients in advance. In comparison to women with healthy pregnancies, females who later developed PE had a higher proportion of pNK cells during early pregnancy (90). However, another assay revealed that the increased proportions of pNK cells were critical regulators of postpartum PE, not PE during pregnancy (98). Before delivery in the third trimester, the proportions of CD56^+^ NK cells in the peripheral blood of PE patients were remarkably higher than those of healthy mothers (93). Moreover, the increase in pNK cells in the early-onset PE population was more than that in the late-onset PE population (92). Consequently, it appears that the count or proportion of circulating NK cells is elevated in patients with or at risk for PE.

**Table 1:** Studies about pNK cells in PE.

Authors	Nationality	Experimental group	Control group	Results	Conclusions
J A Hill et al., 1986 (84)	American	19 PE patients	19 normal primigravidas	The cytotoxicity of pNK cells in PE patients was similar to that of normal pregnant women.	The change of NK cell cytotoxicity seems to have nothing to do with the occurrence of PE.
L Matthiesen et al., 1999 (85)	Swedish	12 PE patients	6 normal pregnant women	The NK cell proportion in severe PE sufferers was remarkably elevated compared with healthy pregnancies.	Elevated number of NK cells was observed in PE cases.
Angela M. Borzychowski et al., 2005 (86)	English	15 PE patients	15 healthy pregnant women	PE patients showed obviously higher NK1/NK2 and NKT1/NKT2 cell ratios compared with healthy mothers.	The type 1/type 2 cell ratio in PE cases was remarkably higher than that in uncomplicated pregnancies.
Angelique L Veenstra van Nieuwenhoven et al., 2008 (87)	Dutch	15 PE nulliparous patients	19 normal pregnant women	The proportion of CD3^-^/CD94^+^ NK cells was profoundly reduced in PE females relative to healthy mothers.	Mild PE women exhibited decreased NK cells compared to those in normotensive pregnant females.
Nora Bachmayer et al., 2009 (88)	Swedish	26 PE patients	28 normal term pregnancies	The expressions of NKG2A and NKG2C on NK cells were obviously increased in PE females.	The receptors on NK cells in PE females’ peripheral blood were changed and affected by IL-12 and IL-15.
J C Bueno-Sánchez et al., 2013 (89)	Colombian	11 early-onset severe PE without HELLP	11 normal pregnant women	The proportion of NK cells having NKG2A or NKG2C and the cytotoxicity of NK cells were statistically increased in severe PE patients.	The function of NK cells in women with early-onset severe PE without HELLP was enhanced, including cytokine secretion, cytotoxic activities and NKG2 expression.
Antonio Simone Laganà et al., 2017 (90)	Italian	13 PE patients	26 normal pregnant women	CD16^+^CD45^+^CD56^+^ NK cells were obviously enhanced in PE cases compared with healthy pregnancies in early pregnancy.	Increased circulating NK cells in the first trimester help predict high-risk groups that may suffer from PE.
Marie-Therese Vinnars et al., 2018 (91)	Swedish	13 PE patients	17 normal pregnant women	The cytotoxic activities of pNK cells were elevated in PE group.	The impairment of villous tissues in PE women may be associated with the increase of local cytotoxicity *via* the NKG2D receptor-ligand interaction.
Mingyue Du et al., 2020 (92)	Chinese	14 early-onset and 28 late-onset PE patients	18 normal pregnant women	The number of pNK cells in PE cases was remarkably higher than that in healthy controls. The percentage of pNK cells in early-onset PE cases was remarkably higher than that of late-onset PE women.	NK cells were involved in the pathophysiology of PE in late pregnancy.
Kimberly Seamon et al., 2020 (93)	American	5 PE patients	5 normotensive pregnant women	The cytotoxicity and stimulation of pNK cells in PE cases were remarkably higher than those in normal pregnancies.	Altered lymphocyte stimulation may be linked with the ERAP1/ERAP2 expression.

**Figure 2:**
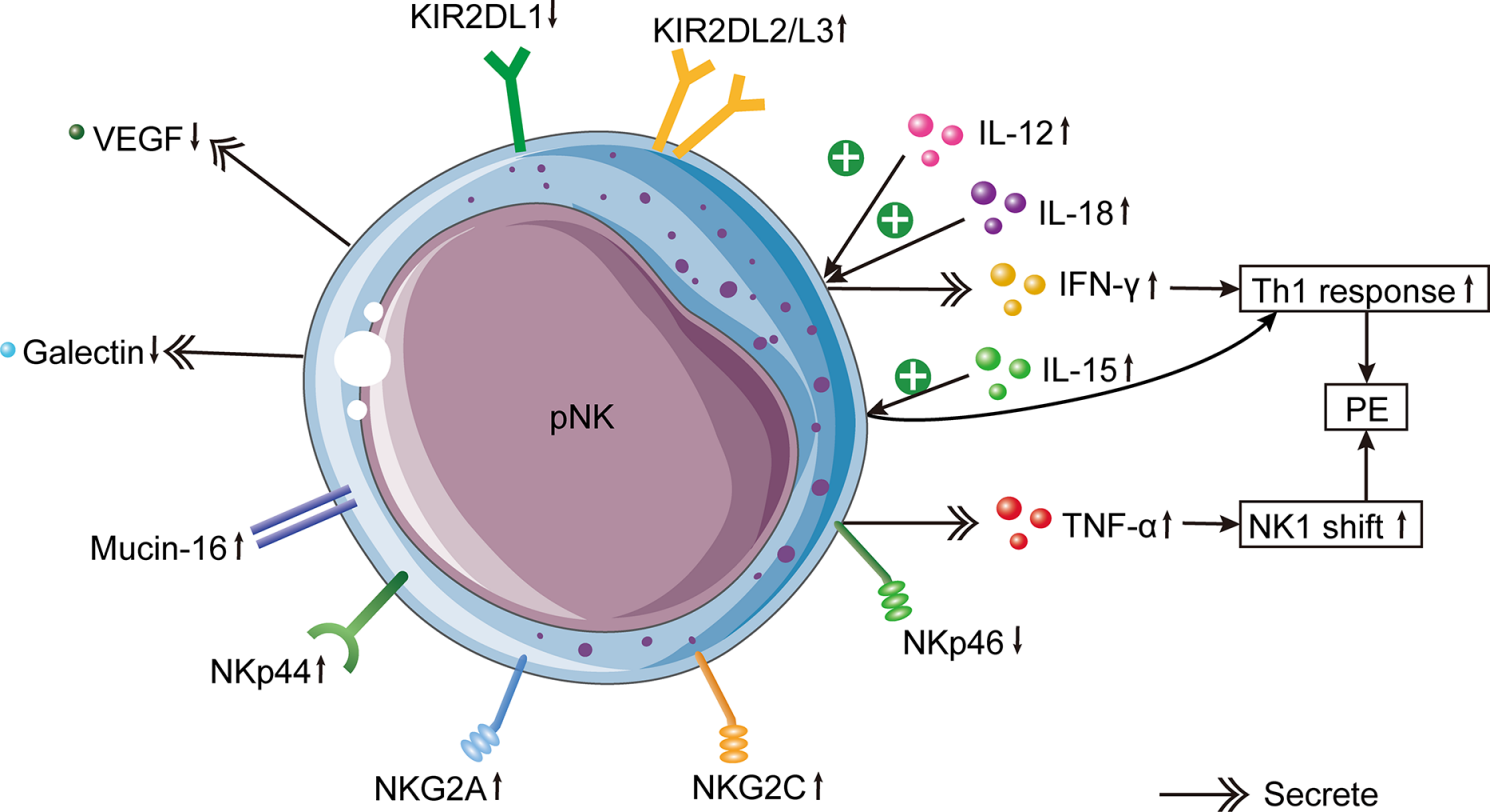
Changes of pNK cells in PE. In PE patients, increased circulating pro-inflammatory cytokines IL-12 and IL-18 induce IFN-γ production by pNK cells, which leads to the Th1 immune bias of PE. In addition, elevated circulating IL-15 induces the systemic inflammatory response by activating pNK cells. pNK cells from PE cases produce less VEGF and more galectin. Mucin-16 has a stronger binding with pNK cells in PE. The inhibitory NKG2A, activating NKG2C and NKp44 in pNK cells of PE patients are increased. Besides, KIR2DL1^+^ NK cells are decreased, whereas KIR2DL2/3^+^ NK cells are increased in PE. In females with PE, there is a lower expression of NKp46 on pNK cells, which causes the increased expression of TNF-α produced by pNK cells. Higher production of TNF-α results in an NK1 shift of NK cells, which is one of the etiologies of PE.

Similarly, several studies have demonstrated that the function of pNK cells is enhanced in PE. In comparison to normal pregnancies, the IFN-γ level secreted by pNK cells was significantly higher in PE patients (99), as was the expression of NKG2A and NKG2C (88, 89). The ratio of type 1 to type 2 NK cells was enhanced in the peripheral blood of patients with PE (86). However, the pregnancy-promoting factors produced by NK cells, such as VEGF (100) and galectin-1 (101), were clearly reduced (**Figure 2**). Mucin-16, or CA-125, is a glycoprotein produced by decidual cells (102, 103). The binding between Mucin-16 and circulating CD16^-^/CD56^bright^ NK cells was stronger in PE patients than in full-term normal pregnant women (104). The greater combination of Mucin-16 and CD16^-^/CD56^bright^ NK cells may reduce these cells’ ability to secrete angiogenic cytokines, resulting in poor remodeling of spiral arteries and insufficient invasion of trophoblasts, leading to PE. In summary, these results show that circulating NK cells in patients with PE are dysfunctional and activated.

## 5 dNK cells in PE

Although the immune cell mechanism in decidua has been studied for many years, the function of dNK cells in PE remains unknown (**Table 2**). A few experiments have demonstrated a rise in the NK cell proportion in the gestational tissues of PE patients (92, 114, 116). During delivery, the number of CD56^+^ and CD94^+^ cells in PE placentas was remarkably higher, while placental IL-12 expression was significantly lower compared to healthy pregnancies (108). Researchers examined the composition of leukocytes in the decidua of PE patients and discovered that dNK cells were greatly increased in PE relative to those from normal pregnant women (54). Similarly, the CD16^+^/CD56^+^ NK cell proportion was remarkably enhanced in the decidua of PE patients (106). Patients with PE had abundant dNK cells with increased CD56 density in their decidua, showing that most dNK cells in PE were in a resting immature state (114). The increased production of CD56^+^ dNK cells in PE creates a decidual microenvironment that captures dNK cells by distorting their conversion and trafficking. The increased number of dNK cells, cytolytic enzymes, and intracellular inflammatory cytokines may inhibit trophoblast migration and invasion, constrain spiral artery remodeling, kill trophoblast cells, and ultimately initiate PE.

**Table 2:** Studies about dNK cells in PE.

Authors	Nationality	Experimental group	Control group	Testing methods	Results	Conclusions
T Stallmach et al., 1999 (54)	Swiss	15 PE patients with IUGR and 14 PE patients without IUGR	19 normal pregnant women	IHC	CD56^+^ dNK cells remarkably went up in PE patients with IUGR compared to healthy pregnant women. There was no significant elevation of dNK cells in PE without IUGR compared with the normal controls.	PE with IUGR may be associated with the increased CD56^+^ dNK cells.
Jacek R Wilczyński et al., 2002 (105)	Polish	21 PE patients	11 normal pregnant women	Flow cytometry	Compared to normal pregnant women, the number of typical CD3^–^/CD56^+^16^+^ cells in PE group was elevated.	The proportion of classical NK cells in decidua of PE patients was increased.
Jacek R Wilczyński et al., 2003 (106)	Polish	21 PE patients	11 normal pregnant women	Flow cytometry	An elevation of CD3^-^/CD56^+^CD16^+^ cells was observed in PE cases compared to healthy expectant mothers.	The alternation of NK cells and the subsequent over-activation of Th1 cytokine IFN-γ may disrupt the immune balance in PE decidua.
Irina P Eide et al., 2006 (107)	Norwegian	8 PE, 5 IUGR, and 17 PE with IUGR patients	31 normal pregnant women	Immunostaining	The experimental group were characterized with obviously lower NK cell percentages compared to the normal controls.	NK cells were associated with the etiology of PE.
Nora Bachmayer et al., 2006 (108)	Swedish	22 PE patients	24 normal pregnant women	IHC	The numbers of CD56^+^ and CD94^+^ cells in decidua of PE sufferers were remarkably higher than those of healthy pregnant females.	The modulation of dNK cells in symptomatic PE was impaired. dNK cells are fundamental for a normal pregnancy.
Paula J Williams et al., 2009 (109)	British	12 PE patients	12 healthy pregnant women	IHC	Compared to normal pregnancies in the third trimester, the CD56^+^ dNK cell number in placental bed biopsies of PE patients was reduced.	The lower number of CD56^+^ dNK cells caused the imbalance of cytokines, resulting in the shallower trophoblast invasion and abnormal spiral artery remodeling.
Lorenz Rieger et al., 2009 (110)	German	33 PE patients	66 normal pregnant women	Flow cytometry	The count of CD56^+^/CD16^+^ NK cells in decidua of PE cases was remarkably less than that of the control group.	The decreased number of CD56^+^/CD16^+^ dNK cells may form a milieu that was detrimental to placental formation, participating in the progression of PE.
Elly N Sánchez-Rodríguez et al., 2011 (111)	Mexican	9 PE patients	10 normal pregnant women	Flow cytometry	The dNK cell number in decidua of PE sufferers was similar to that of the control group.	There were dNK cells in decidua of both groups in the third trimester, which was similar to the cell phenotype in the first trimester.
Charles J Lockwood et al., 2013 (112)	American	30 PE patients	90 normal pregnant women	Flow cytometry	PE pregnancies were characterized with lower CD56^bright^CD16^-^ NK cells relative to healthy mothers.	The enhancement of decidual inflammatory response in the first trimester suppresses the recruitment of pNK cells, causing the lower presence of dNK cells.
Jelena Milosevic-Stevanovic et al., 2016 (113)	Serbian	30 PE patients	20 normal pregnant women	IHC	The proportion of CD56^+^ NK cells in pre-eclamptic decidua was remarkably less than that of healthy expectant mothers. However, the dNK cell number was not related to the severity of PE.	The percentage of CD56^+^ NK cells was associated with the depth of trophoblast invasion, suggesting that they contributed to the pathogenesis of PE, but the relationship between their count and the severity of this disease was not demonstrated.
Jianhong Zhang et al., 2019 (114)	Caucasian, Asian, Black and other population	61 PE patients	26 preterm and 23 term normal pregnant women	Multi-parameter flow cytometry	The count of CD56^+^ CD3^-^ dNK cells in PE women was remarkably increased than that in premature birth or normal full-term pregnancies.	The elevated number of dNK cells caused pathological changes at the maternal-fetal interface, which lead to the occurrence of PE.
Jianhong Zhang et al., 2021 (115)	Canadian	36 PE patients	31 normal pregnant women	Flow cytometry	The PE group contained decreased numbers of CD16^+^ dNK cells compared with normal mothers.	CD16^+^ dNK cells are important for maintaining a fit pregnancy.
Mingyue Du et al., 2022 (92)	Chinese	14 early-onset and 28 late-onset PE patients	40 normal pregnant women	Flow cytometry	The numbers of dNK cells in patients with early-onset PE and late-onset PE were remarkably higher than those in the control group.	dNK cells in the third trimester are linked with the etiopathogenesis of PE.

Nonetheless, other studies discovered that the count of CD56^+^ NK cells in the decidua of patients with PE was greatly lower than in normal pregnancy (110, 112, 113). In PE patients, for instance, the CD16^+^ dNK cell number greatly decreased, indicating that these cells had a protective effect on normal pregnancy (115). According to an assay, the CD56^+^ NK cell number in the decidua had no connection with the disease severity (113). As the etiology of PE involves systemic vascular endothelial cell response and multiple other factors, the clinical manifestations of PE are not solely related to the dNK cell number. In one study, placental bed biopsies were taken from twelve normal pregnancies, eight intrauterine growth retardation (IUGR) patients without hypertension, and twelve PE patients (109). They discovered that the CD56^+^ dNK cell number in people with PE and IUGR was greatly lower than in healthy pregnant women (109). In another separate experiment, the number of CD56^+^ dNK cells was less in PE with IUGR than in healthy pregnancy (107). These outcomes are unexpected given that PE is typically considered as an inflammatory response (117, 118). Oncology studies have demonstrated, however, that pro-inflammatory status is a necessary condition for cancer cells to invade healthy tissues (119, 120). Since trophoblast cells share many similarities with cancer cells, the decreased invasion of trophoblasts in the placenta may be related to the compromise of these inflammatory cells. In addition, the decreased number of dNK cells may reduce the production of angiogenic factors like VEGF, Ang2 and PlGF, resulting in inadequate spiral artery remodeling (62, 121).

Alternatively, many authors have hypothesized the dNK cell function in PE has shifted rather than their absolute number (122). dNK cells separated from pregnant women who had poor spiral artery recasting were less capable of chemically attracting trophoblasts (122). This phenomenon may be attributable to the decreased cytokines produced by dNK cells, which affected the spiral artery development and contributed to hypertension in pregnant women (123). The function of dNK cells is distinct from their number, and the former appears to be more essential because it determines the biological response.

A study also revealed that it was not significantly different between PE patients and normal pregnancies in the number of CD16^-^/CD56^+^ dNK cells (110). Similarly, another study collected decidual tissues from ten normal pregnant women and nine women who had PE (111). Their results indicated that dNK cells continued to exist during pregnancy, while showing no statistical difference between the two groups in the dNK cell number (111). Moreover, the increase in the proportion of dNK cells during the implantation window did not increase the risk of PE in the index pregnancy (124).

**Figure 3:**
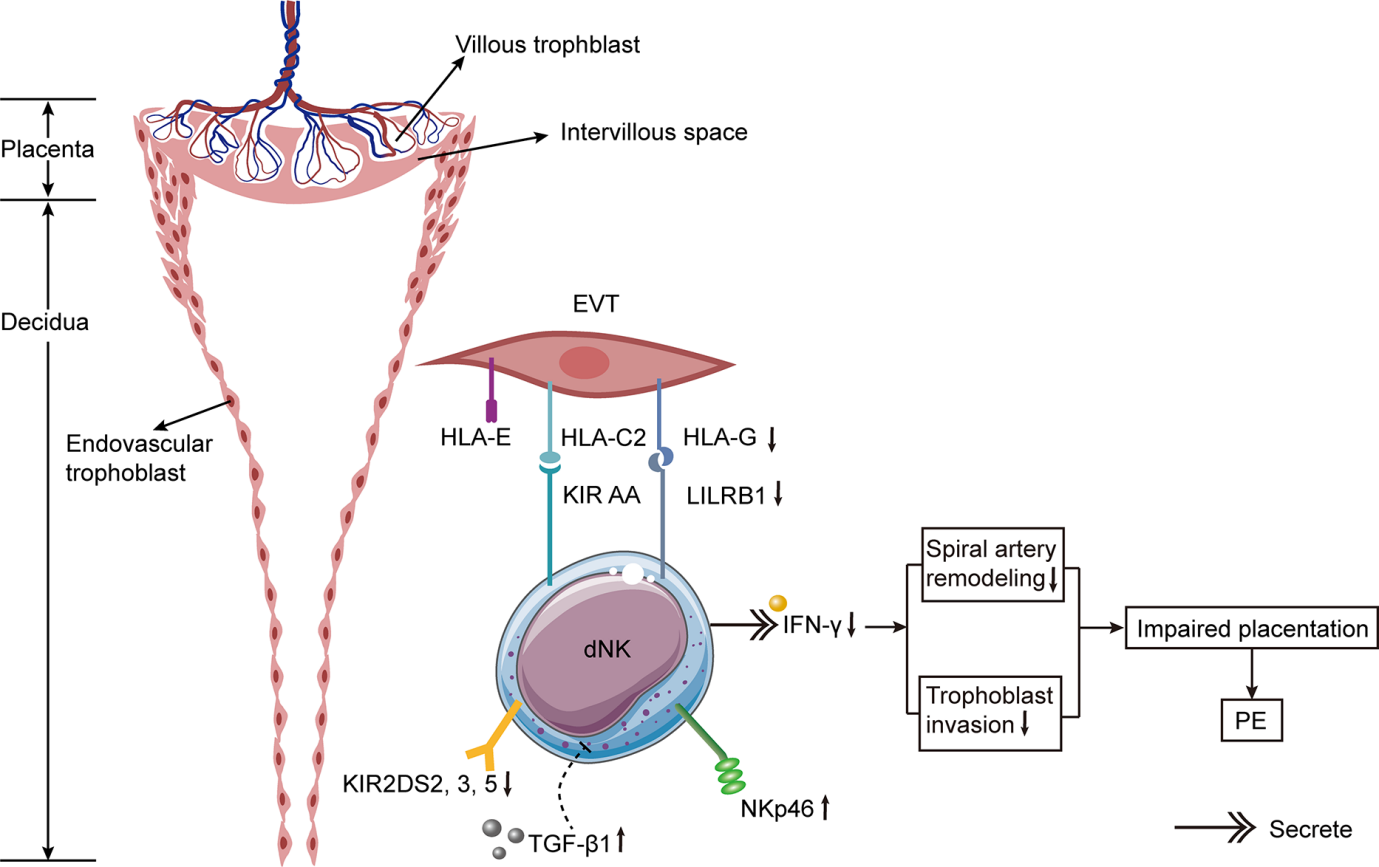
Hypothetical model of alternations of dNK cells in PE. The incidence of PE is significantly increased if the mother has KIR AA genotype and the trophoblast has HLA-C2. The expressions of activating KIR2DS2, 3, and 5 are decreased on dNK cells from PE patients. Besides, the expressions of HLA-G on trophoblast cells and its receptor LILRB1 on dNK cells are diminished in PE. The expression of NKp46 on dNK cells is elevated in PE. The increased expression of TGF-β1 in decidua of females with PE suppresses the activation of specific subpopulations of dNK cell, thus resulting in PE. Lower levels of IFN-γ secreted by dNK cells result in defective spiral artery remodeling and abnormal trophoblast invasion, leading to impaired placentation and ultimately causing PE.

The variation in experimental results may be attributed to altered experimental methods, the use of PE samples with varying disease severity (with or without IUGR), or the distinct position of the collected tissues in the placenta (dNK cells near the spiral arteries or total dNK cells in the placenta). Some studies obtained placental tissues *via* cesarean delivery (106), whereas others obtained placental bed biopsies (54). Due to the invasive nature of placental bed biopsy, the probe may become contaminated with peripheral blood, resulting in changes in the dNK cell number. In one study, researchers obtained decidual tissues from the placental surface after placental delivery or performed vaginal curettage (110). This approach offers minimal contamination from maternal peripheral blood and may represent the true *in vivo* state. Even though the debris on the probe was meticulously cleaned, it is not possible to rule out the possibility of peripheral blood contamination. Besides, the detection method of dNK cells by fluorescence activated cell sorting (FACS) (110) or immunohistochemistry (IHC) (113) was another factor that affected the results. Both approaches have advantages and disadvantages: FACS has more advantages in the precision of quantitative detection, and it can detect a greater number of cells with less interobserver bias; IHC reveals the distribution of various cell types in tissues and can reveal the interaction between cells. Specifically, the percentages of decidual CD56^+^ NKp46^+^ cells were elevated in PE, whereas the percentages of NK cells labeled with other receptors (NKp44, NKp80, or NKG2D) were not increased (114) (**Figure 3**). Due to the different etiology of early-onset and late-onset PE, the increase in pNK and dNK cells in early-onset PE was more pronounced than in late-onset PE when compared to healthy full-term pregnancies (92). The count of dNK cells in IUGR patients (with or without PE) was remarkably lower than in healthy pregnancies (107, 109) and was associated with defective spiral artery remodeling (107).

Both rats and mice are the most popular animal models for examining the dNK cell function in PE because they can be used to examine dNK cells at various pregnancy stages. In an *in vitro* experiment, it was determined that dNK cells in the mesometrial triangle of the PE mouse model decreased significantly during the second trimester, and this decrease was associated with the malfunctioning spiral artery recasting (76, 125). Another PE rat model revealed a correlation between decreased trophoblast invasion and spiral artery recasting and the lower dNK cell count (76). In a separate experiment using BPH/5 mice as PE models, researchers found that the proportion of dNK cells was reduced (126). It has been confirmed that an augment in cytolytic NK (cNK) cells in the placenta of PE rats results in hypertension and growth retardation in their offspring (127). This study demonstrated that Reduced Uterine Perfusion Pressure (RUPP) promoted the activation and proliferation of cNK cells, and that NK cell removal ameliorated hypertension, fetal growth restriction, and inflammation (127). Adoptive transfer of NK cells from RUPP to normal pregnant rats resulted in NK cell stimulation, increased uterine artery resistance index (UARI) and oxidative stress reaction in the placenta, which ultimately caused PE and IUGR (128). RUPP acceptors of NK cells from normal pregnant rats exhibited normal NK activation, plasma soluble fms-like tyrosine kinase receptor-1 (sFlt1), plasma and decidual VEGF, and normal UARI, thereby compromising hypertension and fetal growth restriction (128). The studies point out that cNK cells matter in the pathogenesis of PE, while normal NK cells alleviate hypertension and promote fetal development (128). Compared to removing NK cells from healthy pregnant rats, removing NK cells from PE rats decreased trophoblast cell invasion, but did not affect spiral artery recasting (129). Consequently, similar to the findings in humans, the number of dNK cells in animal models of PE decreased, and their functions may have been altered. More PE model experiments on dNK cells are necessary.

## 6 Cytokines associated with NK cells in PE

The Th1/Th2 equilibrium in PE has gradually shifted to an NK1/NK2 equilibrium based on cytokine production (130, 131). Compared with healthy pregnant females, the ratio of NK1/NK2 cells in the peripheral blood of patients with PE was significantly higher (86). NK cells from PE patients were more cytotoxic than those from normal pregnancies and produced more tumor necrosis factor (TNF)-α and IFN-γ (132). Both cytokines were greatly activated in patients with PE and rats with RUPP (99, 127, 133). The administration of TNF-α to pregnant rats increased mean arterial pressure and vascular resistance in the kidney (134). In rats with placental ischemia, the removal of NK cells reduces blood pressure by inhibiting TNF-α signaling. In addition, IFN-γ is an essential contributor to the etiology of PE caused by cNK cells and may be used as a therapeutic target to alleviate PE symptoms (135). Significantly elevated IFN-γ was secreted by NK cells in the peripheral blood of women with severe PE, and this elevation was associated with syncytiotrophoblast microparticles from the placenta (136). Nevertheless, the level of IFN-γ secreted by dNK cells in PE patients was remarkably less than in regular pregnancies, indicating that the dNK cell function was markedly diminished (114). In the third trimester, IFN-γ activity was greatly enhanced in the decidua of patients with PE, which altered the local immune response (106).

Increased production of IL-12 and IL-18 causes a systemic inflammatory response, which in turn induces NK cell stimulation and increases IFN-γ expression, thereby altering the favorable Th2 immune response into the Th1 response prevalent in PE (86). Consistently, the serum expression of IL-12 was higher in pre-eclamptic patients than in healthy mothers (108, 137). Nonetheless, the reduced expression of IL-12 in the placentas of PE patients indicated that the regulatory function of NK cells was compromised (105, 108). In addition, decidual macrophages produce IL-15, which is essential for the differentiation, proliferation, cytotoxicity, surface receptor expression, and cytokine secretion of dNK cells (138, 139). The elevated IL-15 expression in the peripheral blood of PE women suggests that PE is in a pro-inflammatory state (108). However, the placental expression of IL-15 in PE did not differ significantly from that in healthy pregnancy (108). Moreover, the NK cell depletion decreased the expression of IL-17 in peripheral blood, indicating that placental ischemia may stimulate cNKs to produce IL-17 (140). This result is similar to an earlier study indicating that NK cells in patients with PE are capable of producing IL-17 (140).

TGF-β1 can transform CD56^dim^ CD16^+^ NK cells into CD56^bright^ CD16^-^ NK cells, completing the NK cell transformation (141, 142). Moreover, TGF-β1 was reported to impair the function of CD56^+^ dNK cells (114). dNK cells with low cytotoxicity modulate vascular remodeling by secreting TGF-β1 (62, 143, 144). An experiment with rodents demonstrated that inhibiting the TGF-β1 signal slowed the maturation of NK cells (145). Consequently, TGF-β1 is a primary factor in modulating the function of dNK cells and promoting maternal-fetal immune tolerance. The TGF-β1 expression in the decidua of females with PE was higher than that of females with normal pregnancies, and the increased TGF-β1 expression inhibited the stimulation of certain subsets of dNK cells, thereby causing PE (114). TGF-β1 levels in the circulation and cord blood of late-onset PE patients were greatly lower than those of normal controls, but they were similar to those in early-onset PE cases (146). In addition, one study found that the TGF-β1 expression level in placental tissue of PE cases was remarkably higher than that of healthy pregnant females (147), whereas another research discovered that the TGF-β1 expression level in serum did not differ greatly between PE and fit mothers (148). These discrepancies may be attributed to the unique experimental samples, the racial and ethnic diversity of the subjects, and the limited number of patients in some assays. Additionally, it is barely specified whether the active TGF-β1 was tested in these experiments. Consequently, these results must be evaluated with caution. It is necessary to establish a unified detection standard for TGF-β1 and conduct a large-scale study to determine whether TGF-β1 is associated with the occurrence of PE. In addition, angiogenic factors secreted by circulating NK cells also changed in PE patients. Significantly fewer VEGF-producing pNK cells were found in PE patients than in fit mothers (100). In response to stimulation, pNK cells changed their phenotype and ceased to produce VEGF, but began to secrete inflammatory factors.

## 7 NK cells receptors and PE

During the proliferative and secretory phases of the menstrual cycle, eNK cells in the uterus express NKp46, while NKp30 and NKp44 are either negative or weakly positive, respectively (42). However, another study found that eNK cells in the endometrium in the secretory stage were positive for NKp46, NKp30, and NKp44 (132). Few studies have been conducted regarding NCRs in the field of reproduction. Females with poor pregnancy outcomes may have an aberrant repertoire of NCRs, indicating that NK cells in this population are dysfunctional. NCR-positive cells are more cytotoxic, and women with reproductive failure may have higher cytotoxic activity. Studies have analyzed the expression of NCRs in circulating NK cells of PE patients. PE patients had significantly fewer CD56^+^/NKp46^+^ cells and CD56^bright^/NKp46^+^ cells than normotensive pregnant females (132). Notably, the decrease of CD56^+^/NKp46^+^ cells occurred three to four months before the onset of PE (132). Consequently, NKp46 can be used as an index to predict PE, similar to sFlt1 and PlGF (149, 150). Besides, the declined expression of NKp46 on NK cells in PE patients increased the production of TNF-α and shifted NK cells to the NK1 phenotype (18).

Various activating or inhibiting receptors are present on the NK cell surface. The bias between activating and inhibitory receptors determines the cytotoxicity of NK cells. In addition to NCRs, there are also CD94/NKG2 receptors that play a role in the pathogenesis of PE. The CD94/NKG2 receptors include the activating receptor CD94/NKG2C and the inhibitory receptor CD94/NKG2A. NKG2D is another activating receptor unrelated to CD94 and owns low homology with NKG2A and NKG2C (151). Combined with HLA-E, the CD94/NKG2A receptor on the surface of dNK cells generates inhibiting signals to prevent cell lysis. Experiments on mice demonstrated that depletion of NKG2A during pregnancy led to defective vascular recasting (152). By sequencing the entire genomes of 7,219 PE patients, researchers discovered a 7% increase in the risk of maternal HLA-B failing to educate NKG2A^+^ NK cells (152). HLA-B could bind with activating KIR2DS1, 2, 3, 5, and KIR3DS1 receptors. Patients with PE had significantly higher levels of inhibitory NKG2A and activating NKG2C in their circulating NK cells than normal expectant mothers or healthy women (88). It appears that NK cells attempt to keep a balanced level of NK-cell receptor expression, even though PE patients are about to experience a systemic inflammatory response and have activated pNK cells. In severe PE cases without HELLP (hemolysis, elevated liver enzyme levels, and a low platelet count) syndrome, the proportion of NK cells containing NKG2A or NKG2C was significantly elevated (89). This indicates that patients with severe PE without HELLP have elevated NK cytotoxicity due to the NKG2 receptor (89). The mRNA expression levels of NKG2D receptor and its ligands were remarkably higher in the placentas of PE patients in comparison to those of normal controls (91). The impairment of chorionic villi detected in PE may be due to the enhanced local cytotoxicity caused by the NKG2D receptor-ligand pathway (91). Alternatively, a strong inflammatory reaction may stimulate this pathway, but the signal cannot be neutralized by regulatory cytokines (91).

## 8 KIR/HLA-C and PE

KIR is a predominant receptor expressed on NK cells, combing with HLA-C on trophoblast cells. Trophoblast cells express HLA-C, HLA-G, and HLA-E. Maternal KIR genes are polymorphic and can be either AA or Bx (153). The KIR AA haplotype lacks the majority of activating receptors, except for KIR2DS4. The KIR Bx haplotype owns most activating KIRs (154). As ligands for KIRs, HLA-C can be categorized into two distinct kinds. HLA-C1 binds to KIR2DL2 and KIR2DL3, whereas HLA-C2 combines with KIR2DL1 and KIR2DS1. C2 is superior to C1 in its ability to combine with KIRs (155).

It has been reported that the incidence of PE is increased if the mother has KIR AA genotype and the fetus inherits the father’s HLA-C2 (156, 157). The combination of KIR AA and HLA-C2 may result in insufficient stimulation of dNK cells, affecting the remodeling of spiral arteries and ultimately leading to PE. In patients with PE, the binding of maternal Bx and fetal HLA-C2 increases the risk of atherosclerosis (158). Having additional HLA-C2 from the father significantly increases the PE risk (159). Through high-resolution KIR phenotyping and genotyping of the single NK cell, researchers discovered that women with KIR2DL1A were more susceptible to PE than those with KIR2DL1B (160). A study conducted in Mexico collected decidua tissues from 10 normal pregnancies and 9 patients with PE (111). They discovered a greater number of inhibitory KIRs in the decidua of PE patients (111). In comparison to normal pregnancies, CD158a^+^ (KIR2DL1) NK cells in the peripheral blood of PE sufferers were lower, whereas CD158b^+^ (KIR2DL2/3) NK cells were higher (161). Moreover, the ratio of CD158a^+^/CD158a^+^ NK cells is linked with a rise in blood pressure (161).

In patients with PE, activating KIRs were found to be reduced (162, 163). The activating KIR2DS1 binding to HLA-C2 protects European and Ethiopian women from PE (156, 157, 164). KIR2DS1 stimulates the activation of dNK cells by enhancing angiogenesis and immune response, thereby promoting a healthy pregnancy (27). However, in Caucasians and Africans, KIR2DS4 and KIR2DS5 play a protective role in PE (165–167). Besides, the expressions of activating KIR2DS2, 3, and 5 were lower in Chinese females with PE relative to those with normal pregnancies (163). The whole number of activating receptors in PE sufferers was significantly lower than in the control group (163).

Since Caucasians tend to carry more HLA-C2 molecules than Japanese, Japanese females who marry or have children with Caucasian men tend to have PE than those with Japanese men. Intriguingly, the findings revealed that the incidence of PE in Japanese women with Caucasian husbands was similar to those with Japanese partners (1.54% *vs.* 2.67%) (162). The peripheral blood of pregnant females and newborns from 259 patients with severe PE or eclampsia and 259 women with healthy pregnancies was collected in a separate study (168). They found that KIR/HLA-C combination and severe PE risk were not associated (168). Accordingly, the association between KIR/HLA-C and PE requires further investigation.

## 9 HLA-G and PE

By combining with KIR2DL4 on NK cells, HLA-G inhibits NK cell cytotoxicity and regulates cytokine production (169). Additionally, soluble HLA-G stimulated the production of IFN-γ by both pNK and dNK cells (170). Since soluble HLA-G stimulated the IFN-γ production by dNK cells, it promoted the reformation of spiral arteries and normal implantation (170). Significantly lower HLA-G expression was detected in EVT of PE patients (171–173). In pre-eclamptic placentas, the expressions of HLA-G and its receptor leukocyte immunoglobulin-like receptor subfamily B1 (LILRB1) were significantly lower than in those of normotensive pregnant females (174). Additionally, the expressions of HLA-G and soluble HLA-G in PE sufferers’ peripheral blood were lower than those of women with healthy pregnancies, indicating that insufficient immune tolerance may be the cause of PE (173). The decreased HLA-G expression increases the propensity of NK cells to damage trophoblast cells. Therefore, the reduction of HLA-G on EVT and the NK cell elimination may restrict the invasion of trophoblasts at the maternal-fetal interface, resulting in the constriction and failed conversion of spiral arteries. Notably, another test revealed that the HLA-G expression in PE decidual trophoblasts was similar to that of normal pregnancy (175). Some researchers believed that placental necrosis in PE was responsible for the decrease of HLA-G (176).

## 10 Uterine artery Doppler and dNK cells in PE

Since the outcome of a pregnancy cannot be predicted in advance, it is difficult to obtain dNK cells during the first trimester. To address this issue, specialists sort pregnancies using Doppler ultrasound of the uterine artery. During a healthy pregnancy, the uterine spiral artery undergoes recasting, resulting in increased maternal blood circulation that supplies the placental bed with low pressure. If spiral artery recasting is defective, the resistance index (RI) of the uterine artery will increase correspondingly. RI of the uterine artery can indirectly represent the degree of spiral artery recasting in early pregnancy (177, 178). Some researchers segregated human pNK cells and transformed them into idNK cells, which resemble dNK cells in function and phenotype (179). After administering idNK cells to pregnant mice with elevated uterine artery RI, the number of RI was reduced and placental perfusion was improved (179). dNK cells from expectant mothers with a normal uterine artery RI induced trophoblast invasion more effectively than those from pregnant females with a high uterine artery RI (180). Additionally, dNK cells from high uterine artery RI induced greater endothelial cell activation and TNF-α production than those from normal RI, indicating that the function of these cells has changed (181). In early pregnancy, the expressions of KIR2DL/S1, 3, 5 and LILRB1 were significantly lower in dNK cells from increased uterine artery RI than in those from normal RI (182). This may result in abnormal binding between trophoblasts and dNK cells (122, 123), as well as changes in the production of mediators by dNK cells (123). Pregnant females with PE may have different variations in the uterine artery Doppler parameters during pregnancy (183). Consequently, the monitoring of uterine artery Doppler can be used to evaluate changes in the number or function of dNK cells in PE patients.

## 11 Treatments of PE targeting NK cells

Regarding the treatment of PE, several studies have demonstrated that anti-inflammatory compounds may alter the NK cell number in peripheral blood or decidua, resulting in effective PE treatment. The addition of anti-inflammatory IL-4 to RUPP rat models cut down the number of NK cells in the placenta (184), as did treatment with 17-hydroxyprogesterone caproate (17-OHPC) (185). In addition, the superoxide dismutase mimic tempol decreased the percentages of total NK and cNK cells in peripheral blood (186). Consequently, these specific anti-inflammatory drugs may be used to improve the symptoms of PE due to their ability to regulate NK cells.

In comparison to normal pregnancies, PE patients have changes in the NK cell number affected by progesterone (18, 28, 132). Indirectly or directly, progesterone can decrease the number, activation, and cytotoxicity of circulating NK cells (51). Moreover, progesterone participates in the dNK cell proliferation and differentiation, influencing placentation and fetal development (51, 187–189). However, there are not much research to support the use of progesterone or 17-OHPC to prevent PE (190). Moreover, 17-OHPC did not reduce the risk of recurrent preterm birth (191). Therefore, the therapeutic role of 17-OHPC in patients with PE is unclear.

Intravenous immunoglobulin (IVIg) reduces NK cell cytotoxicity *via* direct antibody action or immunomodulation (192). First of all, IVIg decreased the number of pNK cells in recurrent pregnancy loss (RPL) and achieved reproductive success (193). In addition, IVIg increased the expression of CD94 on NK cells, thereby inhibiting their activity (194). In another study, 31 patients with RPL or repeated implantation failure (RIF) and 15 fertile women participated (195). Patients in the experimental group had more CD56^+^ CD3^+^ NKT cells than those in the control group (195). IVIg therapy greatly reduced the NKT cell number in the study group (195). It can be concluded that IVIg could reduce the number of NKT cells in RPL or RIF patients, thus promoting a healthy pregnancy. IVIg treatment was effective for patients with RIF (196). Before embryo transfer, researchers measured the number of circulating CD16^+^/CD56^+^ NK cells. Then, seven days before embryo transfer, IVIg was administered to women with elevated CD16^+^/CD56^+^ NK cells (196). Compared to placebo, IVIg infusion significantly improved pregnancy outcomes (196). Therefore, IVIg may be an acceptable treatment for PE patients with abnormal NK cell number or activity.

In recent years, it has been illustrated that intralipid can be used to maintain pregnancy (197). Intralipid is primarily made up of purified soybean oil and egg phospholipids. Clinically, intralipid infusion is frequently used to provide malnourished patients with energy and essential fatty acids. Whether intralipid has an immunoregulatory effect remains controversial (198). Several studies point out that intralipid possesses immune activity (199–201). Similar to IVIg, intralipid was reported to suppress the cytotoxicity of NK cells (200, 201). However, future research is necessary to examine the possibility of intralipid in the treatment of PE patients.

## 12 NK cells in cord blood of PE patients

The maternal immune mechanism of PE is well studied, but there is rarely any research about the effect of PE on neonatal immune system. PE patients exhibit an increased inflammatory response, which may impair the immune function of newborns. Alternatively, because maternal-fetal immune tolerance is interactive, the fetal immune system may contribute to maternal pregnancy complications. The NKp30 expression in cord blood NK cells of PE patients was remarkably elevated, whereas that of NKG2D was marginally decreased compared to normal pregnancies (202). The innate immune system of a pre-eclamptic fetus initiates an inflammatory response in the uterus, which is crucial for the function of neonatal NK cells (202). After binding with their respective ligands, aberrant expressions of NKp30 and NKG2D impair the fine regulation of neonatal NK cells. Another study examined the phenotype of NK cells in the umbilical cord blood of both PE sufferers and fit expectant mothers during the third trimester (203). In PE patients’ cord blood, the ratio of CD56^hi^ CD16^-^ non-activated/regulatory NK cells to CD56^lo^ CD16^+^ activated/effector NK cells was significantly decreased (203). This indicates that the pre-eclamptic fetus has an abnormal immune system (203). In addition, the increased inflammatory response and the stimulation of the alternative complement pathway in the cord blood of PE patients indicate that PE affects the neonatal immune compartment (204). The number of CD3^-^/CD56^+^16^+^ NK cells in PE sufferers’ cord blood was significantly higher than that of normotensive pregnant women (205), which was consistent with another study (206). The increase of NK cells transported from the mother to the fetus during PE can explain the rich NK cells in the cord blood of pre-eclamptic females (205). It has been reported that the mother-to-fetus cell shift is elevated in PE (207). Similarly, there is an increase in fetus-to-mother cell transfer in PE (208–211).

## 13 Conclusions

In this review, we summarize the research progress of NK cells in the etiology of PE. We speculate that the innate immune system, as opposed to the adaptive one, plays an important role in immune regulation in pregnancy. NK cells appear to exert a central effect in both peripheral blood and maternal-fetal interface in the etiology of PE. Even though a previous perception believes that dNK cells are “hazardous” cells, current studies suggest that they are necessary for normal implantation, placentation and fetal growth. Accordingly, dNK cells are considered as “peaceful” cells in pregnancy complications, and they collaborate with other members of the innate immune system to construct a healthy reproductive system. Changes in the number or function of dNK cells may be the cause of PE. Genetic studies based on animal models have shown that dNK cells are involved in spiral artery remodeling and trophoblast invasion at the maternal–fetal interface. The abnormal interaction between dNK cells and trophoblasts or maternal cells may contribute to the progression of PE, but the evidence is insufficient.

Even though significant progress has been made in knowing the function of NK cells, the difficulty of obtaining human samples in the first trimester and the limited extrapolation from animal experiments impede the research of NK cells. In addition, the signal pathway governing the dNK cell function during pregnancy, which can predict the occurrence of PE, is unclear. To maintain immune equilibrium both locally and systemically, it is necessary to take therapeutic measures directed at NK cells. Before developing treatments that target these immune cells, it is important to understand the complex mechanism of interaction between decidual cells, as well as the normal function of dNK cells during the first versus third pregnancies and the contrast between these alternations in PE. To determine the reference interval of cytotoxicity and angiogenesis of dNK cells between healthy pregnancies and PE cases, clinical trials are required. To clarify the mechanism of PE, *in vitro* experiments and animal models of PE may mimic the phenotypic alternations of NK cells observed in clinical patients. In addition, basic research aids in the exploration of new NK cell targets and the development of novel technologies to improve maternal and fetal outcomes of PE. In order to improve the prediction, diagnosis, and treatment of PE, therefore, clinically applicable research results are required. We postulate that a more comprehensive understanding of dNK functions and dNK receptor–regulated communications can make a difference in the beneficial role of these cells in PE. The prospective experiments in other pregnancy complications caused by placental abnormalities are requisite to expand on these observations to initiate a latent NK cell-based method to prevent or treat PE and other relevant diseases of impaired placentation.

## Author contributions

XW and XY contributed to the writing of this review. XY designed and revised the manuscript. All authors contributed to the article and approved the submitted version.
